# A Peptoid Delivers CoQ-derivative to Plant Mitochondria via Endocytosis

**DOI:** 10.1038/s41598-019-46182-z

**Published:** 2019-07-08

**Authors:** Kinfemichael Geressu Asfaw, Qiong Liu, Jan Maisch, Stephan W. Münch, Ilona Wehl, Stefan Bräse, Ivan Bogeski, Ute Schepers, Peter Nick

**Affiliations:** 1 Molecular Cell Biology, Botanical Institute, Karlsruhe Institute of Technology (KIT), Fritz-Haber-Weg 4, D-76131 Karlsruhe, Germany; 2 Institute of Organic Chemistry, Karlsruhe Institute of Technology (KIT), Fritz-Haber-Weg 6, D-76131 Karlsruhe, Germany; 3 Molecular Physiology, Institute of Cardiovascular Physiology, University Medical Center, Georg-August-University, 37073 Göttingen, Germany; 4 Institute of Toxicology and Genetics (ITG), Karlsruhe Institute of Technology (KIT), Hermann von Helmholtz Platz 1 D-76344, Eggenstein-Leopoldshafen, Germany; 5 Institute of Functional Interfaces (IFG), Karlsruhe Institute of Technology (KIT), Hermann von Helmholtz Platz 1, 76344 Eggenstein-Leopoldshafen, Germany

**Keywords:** Genetic models, Endocytosis, Plant transporters

## Abstract

Controlled delivery of molecules interfering specifically with target activities in a cell of interest can be a powerful tool for experimental manipulation, because it can be administered at a defined time point and does not require genetic transformation, which in some systems is difficult and time consuming. Peptides as versatile tools that can be tailored for binding numerous binding partners, are of special interest. However, their passage through membranes, their intracellular targeting, and their sensitivity to proteases is limiting. The use of peptoids, where cationic amino-acid side chains are linked to nitrogen (rather than to carbon) of the peptide bond, can circumvent these limitations, because they are not cleavable by proteases. In the current work, we provide a proof-of-concept that such Trojan Peptoids, the plant PeptoQ, can be used to target a functional cargo (i.e. a rhodamine-labelled peptoid and a coenzyme Q10 derivative) into mitochondria of tobacco BY-2 cells as experimental model. We show that the uptake is specific for mitochondria, rapid, dose-dependent, and requires clathrin-mediated endocytosis, as well as actin filaments, while microtubules seem to be dispensable. Viability of the treated cells is not affected, and they show better survival under salt stress, a condition that perturbs oxidative homeostasis in mitochondria. In congruence with improved homeostasis, we observe that the salt induced accumulation of superoxide is mitigated and even inverted by pretreatment with PeptoQ. Using double labelling with appropriate fluorescent markers, we show that targeting of this Trojan Peptoid to the mitochondria is not based on a passage through the plasma membrane (as thought hitherto), but on import via endocytotic vesicles and subsequent accumulation in the mitochondrial intermembrane space, from where it can enter the matrix, e.g. when the permeability of the inner membrane is increased under salt stress.

## Introduction

Genetic engineering has made it possible to control and manipulate living organisms to an extent that is unprecedented by previous strategies. For plants, exogenous deoxyribonucleic acid (DNA) encoding genes mediating the desired traits, is mainly introduced through particle bombardment or *Agrobacterium*-mediated transformation. However, this approach suffers from several drawbacks: the modulation of the trait of interest is indirect and it requires the fact that the introduced information is properly transcribed and translated into proteins that need to be correctly folded and transported to their site of action. The changes introduced by genetic engineering are therefore, of a scalar nature. As a practical constraint, the regeneration of a transgenic organism from a transformed cell can be a lengthy and cumbersome process, as soon as plants other than the model *Arabidopsis thaliana* are used. Hence, alternative methods of manipulation are desirable, such as systems for direct delivery of protein cargoes. However, in order to interact with their intracellular targets, such cargoes have to pass membranes. Cationic oligopeptides are of interest here, because they seem to promote uptake into the cytoplasm, and can be tailored into cell-penetrating peptides (CPPs) as non-viral delivery vehicles for macromolecules in medical applications (reviewed in^1,2^). While in mammalian systems quite different cargoes, such as proteins, plasmids, peptides, nucleic acids, small interfering ribonucleic acid (siRNA), liposomes and nanoparticles have been delivered successfully (reviewed in^3,4^); in plants, the use of such molecular transporters for the delivery of macromolecular cargoes has remained sporadic. This is often attributed to the presence of a rigid cellulosic wall. In fact, CPPs were reported to be readily taken up into cells, where the cell wall had been removed as shown for protoplasts derived from tobacco suspension cells^5^ or Triticale mesophyll cells^6^. However, the notion of the cell wall as impermeable barrier for peptides might not be appropriate, because it is not only possible to introduce CPPs into pollen which is surrounded by a quite massive cell wall^7^, but even into entire plants of *Arabidopsis*, using leaf infiltration^8^.

Although delivery of active cargoes into plant cells via a CPP carrier has been achieved^7–10^, the mechanism of membrane passage has remained a mystery^9,11^. In animal cells, where CPPs have been investigated very intensively as tools for medical application, there is evidence for various mechanisms of uptake that differ depending on the chemical nature of the CPP carrier, and also depending on the chemical properties of the cargo (reviewed in^10,12^). In addition to direct passage through the plasma membrane, by energy-dependent and energy-independent pathways, also endocytotic uptake exists – here, the membrane passage occurs at the endosomal membrane, or by breakdown of endosome integrity. In addition, a reverted micelle mechanism has been postulated. These mechanisms are not mutually exclusive but can act simultaneously. The conclusion drawn from the work on mammalian systems is that one should be very cautious in generalising results obtained from a particular CPP delivering a particular cargo to a particular cell type - each case has to be investigated individually.

Considering the structural (such as the presence of a rigid cell wall) and molecular (such as the absence of integrins, where they have been lost long way back in evolution) differences between mammalian and plant cells, differences in the uptake of CPPs would be anticipated. Nevertheless, certain aspects seem to be conserved. For instance, a role of actin filaments for uptake has been found, both for cationic peptides into mammalian cells^13^, as well as for the uptake into walled tobacco suspension cells^9^. Conversely, this uptake induced in both cases a remodeling of actin filaments linked with the activity of small GTPases of the Rac type^14,15^, and the involvement of specific binding sites as seen from the failure of uptake into heparosulfan glycan negative mammalian cells^14^, and the saturation of the CPPs uptake in the µM range into walled tobacco cells^15^.

Although CPPs are effective *in vitro*, their bioavailability *in vivo* is limited due to degradation by proteases. Thus, peptide mimetics with elevated stability provide interesting alternatives. For instance, by linking the side chain to the amide nitrogen instead of the α-carbon, the resulting oligo-N-alkyl glycine peptoid would not represent a target to peptidases and should be more stable as compared to a CPP. Moreover, these peptoids lack the hydrogen-bonding potential, which should increase bioavailability due to reduced aggregation that originates from the backbone structure^16^. Due to the presence of structurally diverse amines, it is possible to produce peptoid libraries that can be conveniently recombined in a modular fashion without the need for protecting groups as they are needed in CPPs^17^. Such peptoids have been successfully synthesised and applied as effective, water-soluble, non-toxic molecular vehicles for intracellular drug delivery^16^. Poly-guanidine peptoids readily entered walled tobacco cells^18^ and uptake required actin and microtubules. Based on a modular approach, structure-function relationships of uptake and subcellular localization have been mapped in mammalian cells and whole vertebrate organisms^19^. It was shown that increasing hydrophobicity in addition to the cationic residues is driving the peptoids towards mitochondria. Amphiphilic triphenylphosphonium cations (TPP^+^) and strongly amphiphilic peptides with alternating cationic and aromatic amino acid residues such as the Szeto-Schiller-peptides^20^ are known to enter the mitochondria of mammalian cells. These compounds have even been used to transport molecules with antioxidative potential to the organelle of action, the mitochondria. The most studied representatives of this class are the above-mentioned Szeto-Schiller peptides, containing a tyrosine or a dimethyltyrosine residue as an antioxidant entity. Furthermore, TPP^+^ cations have been used to deliver redox active molecules such as ubiquinone (MitoQ)^21^ or plastoquinone CoQ Derivatives (SKQ1) into the mitochondrial matrix^21,22^.

**Figure 1:**
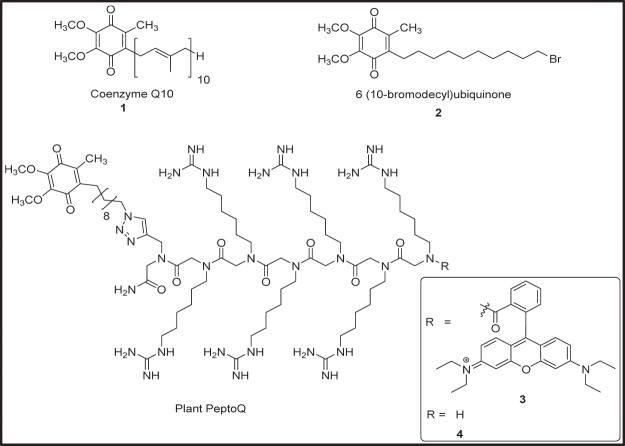
The chemical structures of CoQ10 **1**, the CoQ10 analogue 6-(10-bromodecyl) ubiquinone **2** and the rhodamine-labelled and unlabelled plant PeptoQ **3** and **4**.

In the present study, we extend this strategy to target the mitochondria in plant cells by linking a functional coenzyme Q10 (CoQ10) derivative, where we exchanged the isoprenoid part with an C_10_ aliphatic chain as it was also used for the SKQ and MitoQ (Fig. 1). The chemical composition of plant membranes differs from that in mammalian cells – for instance, cholesterol is replaced by a complex mixture of sterols (for a comprehensive review see^23^). Therefore, more hydrophilic residues are required. We have therefore, tailored PeptoQ especially for the application in plant cells such as tobacco BY-2. As this peptoid is specific for the application in plant cells, it was named as plant PeptoQ. Its isoprenoid-conjugated templates CoQ10 and CoQ1 (for review see^24,25^), and the non-isoprenoid conjugated template, CoQ0^26^, possess remarkably strong antioxidative potential (which is comparable to that of vitamin C) in their hydroxylated forms^25–27^. Note that the presence and the length of the isoprenoid side chain has no impact on the redox properties and antioxidative potential of the above CoQs^28^. Thus, the plant PeptoQ, mimetic of CoQs (which contains similar benzoquinone head structure like them) also acts as an antioxidant and is expected to interact with the electron transport chain from the mitochondrial intermembrane space.

## Results

### Targeting of plant PeptoQ to the mitochondria is a biphasic process

**Figure 2:**
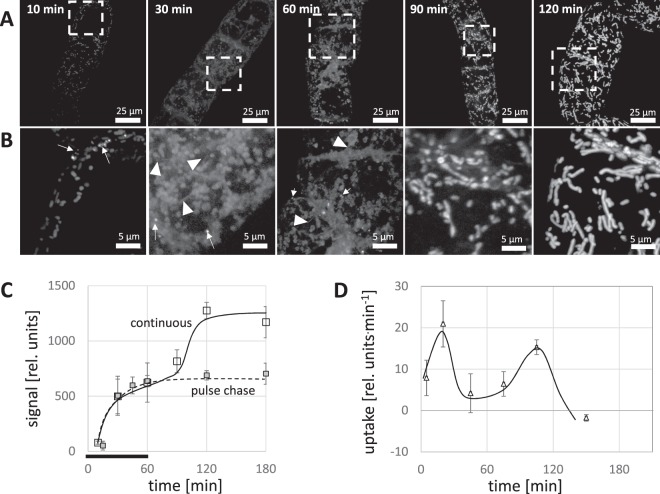
Time course for the uptake of plant PeptoQ into tobacco BY-2 cells. Cells were treated at day 3 after subcultivation (at the completion of the proliferation phase of the culture) with 2 µM of plant PeptoQ and followed by spinning disc confocal microscopy making use of the fluorescent signal of the conjugated rhodamine. The peptoid was present throughout the experiment. Geometric projections of confocal z-stacks collected from representative cells recorded at constant laser power and exposure time are shown in (**A**), The white squares mark the region of interest magnified in (**B**) to show the subcellular details. White arrows indicate punctate structures that are seen in early time points which represent endoplasmic reticulum (ER) structures that have active role in the internalization process, white arrowheads indicate filamentous structures. (**C**) Quantification of intracellular accumulation based on the integrated intensity inside the cell corrected for background either under continuous presence of 2 µM plant PeptoQ (white squares, solid curve) or as pulse-chase, where cells were washed after one hour of incubation with 2 µM and then followed further (grey squares, dashed curve). Mean values and standard errors from three independent experiments are shown. (**D**) Uptake determined from the first derivative of the cumulative time course shown in (**C**) to show the non-steady nature of the process, where a first period of rapid uptake in the first 20 min is followed by a static period, and then the second wave of uptake became evident after 90 min. Beyond 120 min (at 180, 240, 300 and 360 min), uptake became saturated.

Time-course and dose-response experiments were conducted to gain insight into the cellular uptake aspects of the plant peptoQ by walled, non-transformed tobacco BY-2 cells. Uptake was quantified by measuring the mean fluorescence intensity of the plant PeptoQ-associated rhodamine, corrected for background intensity^15^, in proliferating cultures at day 3 after subcultivation^29^. For the time-course study, BY-2 cells were incubated with 2 µM of labelled plant PeptoQ (i.e. the peptoid was present throughout the experiment), and the rhodamine signal was followed by spinning disc confocal microscopy (Fig. 2A). The signal rapidly increased during the first hour of uptake and was then accumulating in vermiform structures that were interpreted as mitochondria. However, during the first hour, most of the signal for this mitochondrial pattern was not developed, which can be visualized by normalising the signal amplitude for the overall lower levels at the early stages of uptake (Fig. 2B). While at 10 min after the start of incubation (the earliest time point that could be reliably observed in this time series), mostly punctate signals were seen in the peripheral cytoplasm, just adjacent to the cell membrane, and at 30 and 60 min of incubation, a reticulate signal was dominating, probably representing the endoplasmic reticulum (ER). Only later, from 90 min of incubation, the mitochondria became evident, and the reticulate signals outside of mitochondria disappeared progressively.

Also, the quantification of the signal over time revealed a non-steady nature of uptake (Fig. 2C). While the uptake was rapid during the first 20 min, it has halted between 30 and 60 min, and then a second wave of uptake became evident between 60 and 120 min. Then, beyond 120 min (at 180, 240, 300 and 360 min), no further uptake was seen (Fig. 2C, white squares, solid curve). When the rate of uptake was plotted over time, the two waves of uptake activity became clearly evident (Fig. 2D). The first wave of uptake coincided with the reticulate distribution of the fluorescent signal, while the second wave of uptake was seen at a time, when the signal from the first wave had been mostly translocated to the mitochondria. Thus, both the cellular details as well as the quantification, show that the plant PeptoQ reaches the mitochondria not in a linear process, but in a biphasic process, where in a first step a pool in the ER is filled, before the signal in a second, slower step accumulates in the mitochondria themselves. To assess, whether the second wave was dependent on renewed uptake, the experiment was modified as pulse-chase, where 2 µM of plant PeptoQ was administered for 1 h and then washed out to follow uptake further. Under these conditions, the intracellular signal remained at the level reached at 1 h, i.e. the end of incubation with plant PeptoQ (Fig. 2C, grey squares, dashed curve).

**Figure 3:**
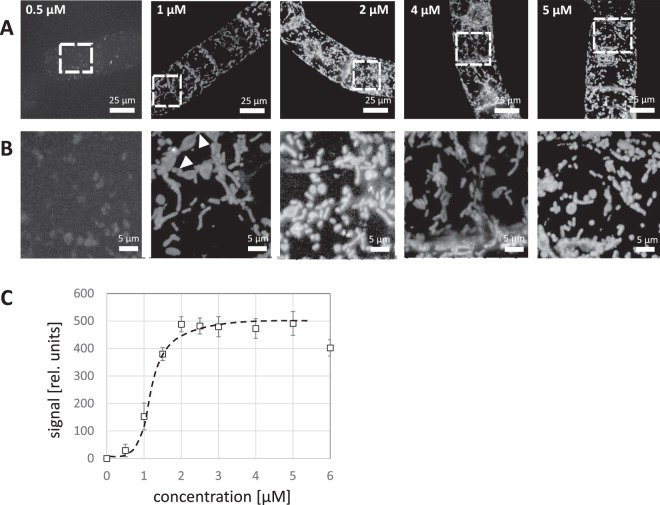
Dose-response for the uptake of plant PeptoQ into tobacco BY-2 cells. Cells were treated at day 3 after subcultivation (at the completion of the proliferation phase of the culture) with 0.5-5 µM of plant PeptoQ, incubated for 2 h, washed three times, and subsequently, observed by spinning disc confocal microscopy making use of the fluorescent signal of the conjugated rhodamine. Geometric projections of confocal z-stacks collected from representative cells recorded at constant laser power and exposure time are shown in (**A**), The white squares mark the region of interest magnified in (**B**), To show the subcellular details. White arrowheads indicate connective structures between mitochondria. (**C**) Quantification of intracellular accumulation based on the integrated intensity inside the cell corrected for background. Data are fitted by a sigmoidal curve. Mean values and standard errors from three independent experiments are shown.

To dissect uptake further, a dose-response study was conducted, where the BY-2 cells were incubated for 120 min (the time point, when uptake was seen to be complete in the time-course experiment) with 0.5 to 5 µM of labelled peptoid. Again, the peptoid was present throughout, and was only washed at the end before microscopical observation. Again, the dependency was not linear (Fig. 3A,B): for 0.5 and 1 µM, the resulting signal was not confined to mitochondria, while for higher concentrations, the mitochondrial signal clearly dominated the background in the cytoplasm. Similar to the situation at early time points seen during the uptake of 2 µM of plant PeptoQ (Fig. 2B), structures with connecting patterns could be seen indicative of a situation, where the peptoids were still trapped in the ER and did not travel further (Fig. 3B, arrow heads). When a dose-response curve was constructed from a quantification of the signals, a significant increase in uptake became evident, when the concentration of the plant PeptoQ was raised from 0.5 to 2 µM. Uptake became saturated above this concentration (Fig. 3C). There was a clear threshold at around 1 µM plant PeptoQ, below which the signal was virtually absent.

The dose-response study is therefore consistent with the conclusion from the time-course experiments that uptake proceeds in two stages. The first stage is rapid leading to an accumulation of the signal in the ER and is saturated at ~2 µM while the second stage proceeds more slowly, shows a higher affinity and distributes the signal from the ER to the mitochondria itself, where it accumulates to high levels.

### Mitochondria are the final destination for the plant PeptoQ

**Figure 4:**
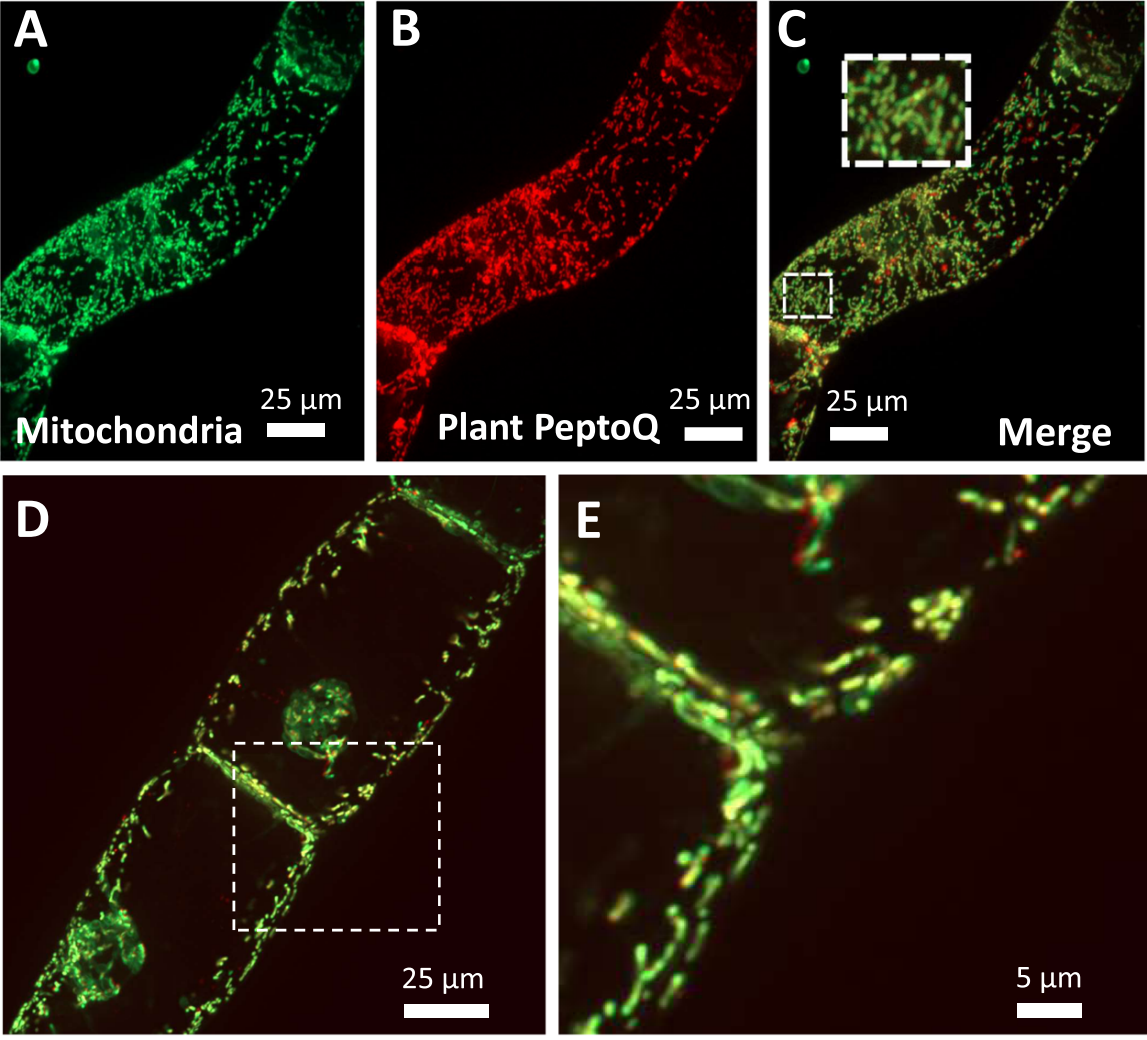
Mitochondrial localization of the plant PeptoQ in tobacco BY-2 cells assessed at day 3 (**A–C**) or day 6 (**D**,**E**). Cells were incubated for 2 h with 2 µM of plant PeptoQ followed by labelling with MitoTracker Green FM and then followed by spinning disc confocal microscopy making use of the fluorescent signal from MitoTracker Green FM (**A**), and the rhodamine signal from the labelled plant PeptoQ (**B**). The merge of the two channels shown in (**C–E**) shows the close overlap of the two signals. Geometrical projections of confocal z-stacks collected from the mid-plane from representative cells recorded at constant laser power and exposure time are shown.

The vermiform-vesicular final pattern obtained for the labelled plant PeptoQ was interpreted as mitochondrial localisation. To test this assumption, cells were treated with 2 µM plant PeptoQ and incubated for 120 min, washed thoroughly, and incubated with the specific mitochondrial dye MitoTracker Green FM for 5 min. The potential co-localization of both signals was then assessed by spinning disc confocal microscopy using simultaneous excitation of the MitoTracker Green FM dye at 488 nm, and the rhodamine conjugated to the plant PeptoQ at 561 nm. The results showed a clear co-localization between the mitochondrial dye and the plant PeptoQ conjugated probe (Fig. 4). This tight association of the signal recording, the plant PeptoQ with mitochondria was observed independently of the proliferation status and was seen both for cells from the proliferating (Fig. 4A–C) or the stationary (Fig. 4D,E) phases of the culture.

### Endocytosis is necessary for the cellular uptake of the plant PeptoQ

**Figure 5:**
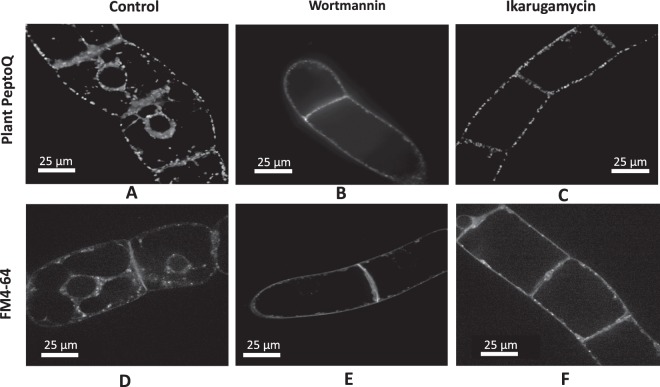
Inhibitors of endocytosis impair the cellular uptake of plant PeptoQ into BY-2 (**A–C**) as compared to the endosomal tracer FM4-64 (**D–F**). Control cells (**A**,**D**) are shown in comparison to cells that had been pretreated for 30 min with either 33 µM Wortmannin (**B**,**E**) or with 10 µM Ikarugamycin (**C**,**F**). Confocal sections recorded in the mid-plane from representative cells are shown.

To test whether uptake was dependent on endocytosis, we used two inhibitors known to disrupt endocytosis: Wortmannin (Wm) blocks phosphatidylinositol 3-kinase, which disrupts the receptor-dependent transport from the plasma membrane to the trans-Golgi network^30^, possibly linked with the activity of dynamin-related protein (for a discussion, refer to^31^). Ikarugamycin (IKA) specifically inhibits clathrin-dependent endocytosis, while allowing for clathrin-independent endocytosis to proceed^32^. Also, in tobacco pollen tube^33^ and protoplasts from tobacco cells^34^, this inhibitor was found to suppress only a part of endocytotic uptake. To verify the effectiveness of these drugs, the uptake of the fluorescent endosomal marker FM4-64 (2 µM, 5 min) was used. While in control cells, FM4-64 was readily taken up (Fig. 5D), such that numerous endosomes could be seen in trans-vacuolar strands and also around the nucleus, a pretreatment with Wortmannin (33 µM) prevented FM4-64 from being internalized (Fig. 5E). Interestingly, the residual signal was confined to the cross wall. Thus, in our system, Wortmannin effectively blocks endocytosis. Then, tobacco BY-2 cells of the same stage (day 3 after subcultivation) were pretreated with Wortmannin for 30 min, and further incubated with 2 µM of the plant PeptoQ for additional 2 h, a time that otherwise would allow uptake to be completed (Figs 2, 5A). Similarly, to the FM4-64 experiment, uptake of the plant PeptoQ was blocked efficiently (Fig. 5B). Again, residual signals were detectable only along the cross wall. To determine, to what extent the endocytotic uptake of the plant PeptoQ depends on clathrin, we used Ikarugamycin. First the efficiency of Ikarugamycin on the uptake of FM4-64 was assessed. Pretreatment with 10 µM Ikarugamycin for 30 min eliminated (Fig. 5F) the fluorescent endosomes in trans-vacuolar strands and around the nucleus that were found in the control (Fig. 5D). Instead, punctate signals were lining the membrane like beads on a string. It should be noted that this signal was not confined to the cross walls, unlike the situation created by treatment with Wortmannin (compare Fig. 5E,F). Then, the effect of pretreatment with Ikarugamycin was assessed for subsequent incubation with plant PeptoQ for 2 h. Here, the pattern was the same as seen for FM4-64, a punctate rhodamine signal was seen at the cell membrane, predominantly at the lateral walls (Fig. 5C). It should be noted that hardly any mitochondria were detectable, despite the 2 h of incubation with the plant PeptoQ, a time interval that otherwise would allow for complete uptake.

**Figure 6:**
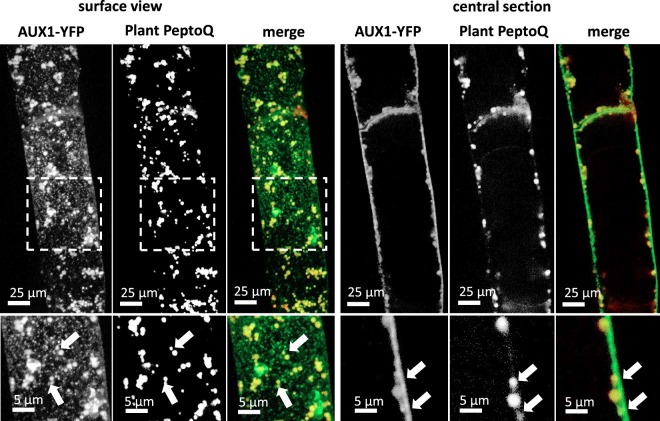
Colocalization of a YFP fusion with the auxin-influx carrier AUX1 (as marker for endosomes) with the plant PeptoQ in surface view (left-hand panel) and in a central confocal section (right-hand panel). White arrows indicate vesicular structures that are labelled by both signals. Expression of AUX1-YFP was driven by an estradiol-driven promoter, which was induced 24 h prior to the experiment by beta-estradiol (1 µM).

To test whether the plant PeptoQ passes through endosomes, the plasma membrane-located auxin-influx carrier AUX1 was employed. A tobacco BY-2 strain expressing a fusion of AUX1 with yellow fluorescent protein (YFP) under an estradiol-driven promoter^35^ was induced for expression and then incubated with the plant PeptoQ. Upon inspection of the cell surface (Fig. 6, left-hand panel), fine punctate signals of AUX1 could be observed that were aligned like beads on a string. These strings were interspersed with larger speckles that presumably represented sites of endocytotic activity and often were clustered to larger agglomerations. The plant PeptoQ signal was seen in these speckles and in these agglomerations, not in the aligned *punctae* that probably represent the working form of the AUX1 carrier. In confocal sections of the cell center, the topology of uptake could be observed (Fig. 6, right-hand panel). – here the plant PeptoQ was seen in vesicular structures of different size, whereby the size of the small vesicles was close to the diffraction limit, while others were larger.

### Actin filaments are important for the cellular uptake of the plant PeptoQ

**Figure 7:**
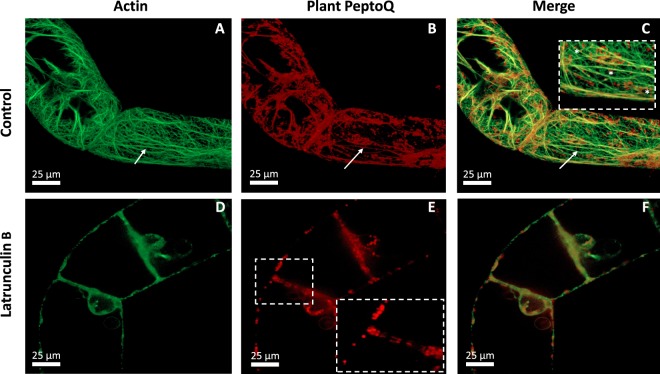
Uptake of plant PeptoQ depends on actin. Cells of the actin marker line GF11 cells (collected at day 3 after subcultivation) were incubated with 2 µM of plant PeptoQ for 2 h either without (**A–C**) or with (**D–F**) elimination of actin filaments by Latrunculin B (10 µM, 1 h). The GFP signal recording actin (**A**,**D**), the rhodamine signal recording the plant PeptoQ (**B**,**E**), and the merged signal (**C**,**F**) is shown in a central confocal section of representative cells. The inset in (**C**) highlights the association of the rhodamine-labelled mitochondria with actin filaments (asterisks), the white arrow in (**A–C**) the association of the rhodamine signal along actin, the inset in (**E**) the signals lining the two sides of the cross wall after treatment with Latrunculin B.

To gain insight into the role of actin filaments in the cellular uptake of the plant PeptoQ, we used the marker line GF11, where actin filaments are visualised by a fusion of the actin-binding domain 2 of *Arabidopsis thaliana* fimbrin with Green Fluorescent Protein (GFP) as reporter. Following a treatment with 2 µM labeled plant PeptoQ for 2 h, we assessed the localization by spinning disc confocal microscopy using the signal from the conjugated rhodamine to see the relation with actin filaments, labelled by the Green Fluorescent Protein (Fig. 7A–C). This dual labelling not only revealed that the mitochondria visualised by the plant PeptoQ were often aligned along actin filaments (Fig. 7C, inset, asterisks), but also that the rhodamine fluorescence signal was partially superimposed (within the limits of resolution) with that of GFP-labeled actin filaments (Fig. 7B, indicated by an arrow). To test, whether actin is important for uptake, the GF11 cells were pretreated for 1 h with 10 µM of Latrunculin B, a specific inhibitor that sequesters G-actin from assembly into F-actin. This treatment was sufficient to completely eliminate actin filaments (Fig. 7D). When these pretreated cells were incubated with 2 µM of the plant PeptoQ, the internalization of the signal was strongly impaired (Fig. 7D,E): only few punctate signals lining the nuclear envelope were observed. It should be noted that the nuclei were shifted to the cross walls. Further signals were lining the cell membranes at the lateral walls (similar to the situation observed for treatment with Ikarugamycin, (compare Figs 7D and 5F). At the cross walls, occasionally a double line could be detected, presumably representing the cell membranes of the two neighbouring cells (Fig. 7E, inset). These observations show that actin filaments are required for the mitochondrial localization of the plant PeptoQ.

### Microtubules are not involved in the cellular uptake of plant PeptoQ

**Figure 8:**
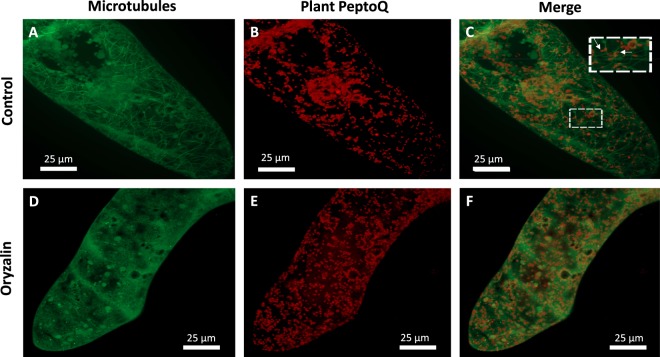
Uptake of plant PeptoQ does not depend on microtubules. Cells of the microtubule marker line AtTuB6 (collected at day 3 after subcultivation) were incubated with 2 µM of plant PeptoQ for 2 h either without (**A–C**) or with (**D–F**) elimination of microtubules by Oryzalin (10 µM, 1 h). The GFP signal recording tubulin (**A**,**D**), the rhodamine signal recording the plant PeptoQ (**B**,**E**), and the merged signal (**C**,**F**) is shown for representative cells. The inset in (**C**) highlights cases, where the rhodamine-labelled mitochondria deviate in their orientation from the microtubule (arrows).

By analogy to actin filaments, a potential role of microtubules for the internalization of the plant PeptoQ was addressed using a cell line expressing the beta-tubulin TuB6 from *Arabidopsis thaliana* fused with the Green Fluorescent Protein. To eliminate microtubules, cells were pretreated with 10 µM Oryzalin, a plant-specific inhibitor of microtubules, for 1 h, before incubation with 2 µM plant PeptoQ for additional 2 h. In the control, without Oryzalin pretreatment, cortical microtubules were clearly seen against the background (Fig. 8A), which presumably comes from non-assembled tubulin heterodimers. When the microtubule signal was overlayed with the rhodamine signal from the labelled plant PeptoQ (Fig. 8B), at first glance, the merged figure (Fig. 8C) resembles that seen for actin. However, a closer look reveals two important differences: While some mitochondria co-localize with microtubules, often they are oriented in a deviating orientation (Fig. 8C, white arrows in the inset). Pretreatment with Oryzalin eliminated microtubules completely, while the fluorescent background signal was now more prominent (Fig. 8D). Although microtubules had been removed by Oryzalin, the pretreated cells were able to take up plant PeptoQ effectively (Fig. 8E,F). Thus, microtubules are not involved in the cellular uptake of plant PeptoQ.

### Plant PeptoQ does not cause any toxicity but mitigates salt-induced mortality

**Figure 9:**
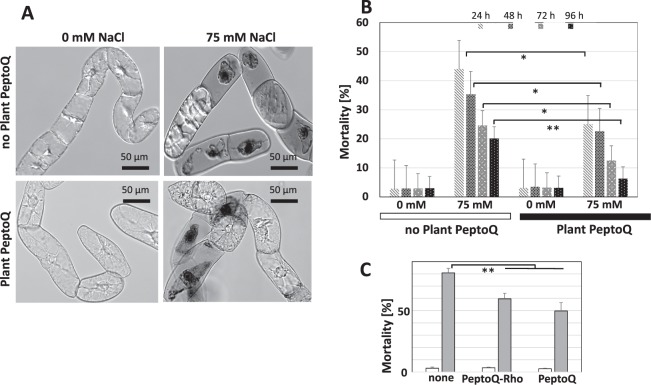
Plant PeptoQ does not cause any toxicity and mitigates the mortality caused by salt stress. (**A**) Representative cells probed by the Evans Blue dye exclusion test. (**B**) Steady-state levels of mortality over time in response to salt stress in presence or absence of plant PeptoQ. (**C**) Effect of rhodamine conjugated to PeptoQ-Rho on the mitigation of salt-induced mortality (steady-state levels) by plant PeptoQ. Data represent means and standard errors from at least three independent experimental series scoring samples of 1000 individual cells. Salt stress (75 mM NaCl) was administered from 2 h after the addition of the plant PeptoQ. ** indicate differences that are significant at *P* ≤ 0.01, *at *P* ≤ 0.05.

A tool for chemical manipulation should not impose toxicity on the target cell. We therefore scrutinized potential toxic effects of plant PeptoQ on BY-2 cells after loading with 2 µM plant PeptoQ for 2 h and then following potential changes of mortality by the membrane-impermeable dye Evans Blue (2.5% w/v). Since dead suspension cells progressively decay after around two days, the Evans Blue assay reflects the steady state level of mortality. We observed that the cells excluded the dye both in control cells as well as in cells incubated with the plant PeptoQ (Fig. 9A), cell toxicity was low (<5%) in both cases and there was no significant difference for the cells treated with the plant PeptoQ, even for prolonged cultivation for 96 h (Fig. 9B). In a supplementary experiment, we followed potential toxicity in response to increasing concentrations of plant PeptoQ (2, 4, 8, 16, 25, 40 and 50 µM) over prolonged time (24, 48, 72, and 96 h) but we could not detect any indication of increased cell toxicity (Suppl. Fig. S1). Mortality was below 5% throughout. To test whether the time frame of up to 96 h was sufficient to pick up potential programmed cell death, we used cell death induced by salinity stress (150 mM) as positive control. Here, the full level of mortality (60%) was already reached after 24 h with only minor increases during the subsequent days (at day 4, around 66% were reached). Since the plant PeptoQ was found to fully preserve the physiology of the treated cells even for prolonged incubation, we asked in the next step, whether a pretreatment with plant PeptoQ could mitigate cellular damage imposed by disrupted oxidative balance in the mitochondria. As stressor, we used salt stress (75 mM NaCl), which induced significant toxicity, accompanied by cytosolic shrinkage, breakdown of cytoplasmic structure, and loss of membrane integrity as visualized by the penetration of Evans Blue (Fig. 9A). The mortality induced by 75 mM NaCl reached almost 50% within 24 h but dropped subsequently over the following days specifically day 4 (96 h) to around 20%, indicative of progressive adaptation and subsequent proliferation of surviving cells compensating for the initial mortality (Fig. 9B). This decrease reflects the adaptation and robust proliferation of the surviving cells. Thus, the decrease of mortality reflects the improved level of adaptation and subsequent recovery in presence of plant PeptoQ. When the cells were pretreated with the plant PeptoQ prior to the salt stress (75 mM NaCl), the initial mortality was much lower (from almost 25% to around 7%), and the subsequent recovery proceeded more rapidly until 96 h after the onset of salt stress, only 7% of cells were found to be dead (at 96 h), which was not significantly different from cells that had not experienced any salt stress (Fig. 9B). This means that the plant PeptoQ not only did not impose any toxicity, but in addition significantly improved the survival and adaptation to salt stress. To test, whether this mitigation of salt-stress induced mortality is linked with an antioxidative activity, we measured the accumulation of superoxide in response to salt stress with and without plant PeptoQ pretreatment (Suppl. Fig. S1). We observed that superoxide levels increased in a dose dependent manner in salt-stressed BY-2 cells that were not pretreated with plant PeptoQ, whereas superoxide levels were not only mitigated by plant PeptoQ pretreatment, but even decreased. On the other hand, to test, whether the mitigation of salt-induced mortality was dependent on the presence of rhodamine moiety, we probed mortality under stringent salt stress (150 mM) over two days (Fig. 9C). The unconjugated peptoid seemed to be slightly more effective than its rhodamine conjugate, but the difference was not significant. What was highly significant, however, was the mitigation of mortality in the peptoid treated samples.

### Plant PeptoQ can suppress mitochondrial fragmentation induced by perturbed electron transport

Salinity stress (75 mM NaCl) was administered to BY-2 cells at day 3 after subcultivation either without or with preincubation (for 2 h) with plant PeptoQ, and mitochondrial area coverage was determined 2 h and 24 h after the onset of salt stress. While mitochondria were of ovoid shape under control conditions, they were found to have decayed into small fragments after treatment with 75 mM NaCl (Suppl. Fig. S3A). This effect could be completely mitigated, when the cells were pretreated with plant PeptoQ before the onset of salt stress.

## Discussion

### Plant PeptoQ targets to mitochondria in two steps involving passage through the ER membrane

The signal increased over time which is to be expected, because the peptoid was present throughout during the experiment. To preclude the notion that the increase in fluorescence signal might originate from changes in environmental conditions such as pH, salt or stacking, we conducted a pulse-chase experiment where, BY-2 cells were treated with 2 µM rhodamine labeled plant PeptoQ, incubated for 60 min and finally washed-out three times. The result showed that the signal was levelled off at a lower plateau (Fig. 2C). These observations support a model of biphasic uptake. In the first step, the peptoid accumulates in endosomes and ER, followed by a second step, where these intermediate pools are depleted and the peptoid transferred to the mitochondria (Fig. 4). While the outer mitochondrial membrane probably does not represent a barrier due to the presence of porins with an exclusion size limit of 5 kDa (reviewed in^36^), there must be one point, where the peptoid has to pass a membrane to reach the outer mitochondrial membrane. Principally, this membrane passage could occur at the plasma membrane. Alternatively, the peptoid might be taken up through endocytosis and exit into the cytoplasm from endosomes. Both mechanisms have been reported for the uptake of cell-penetrating peptides into mammalian cells (reviewed in^10,12^) depending on the type of transporter and also depending on the type of cargo. In plant cells, to the best of our knowledge, this is the first case, where the uptake of peptoids into mitochondria has been demonstrated and the mechanism of uptake investigated.

**Figure 10:**
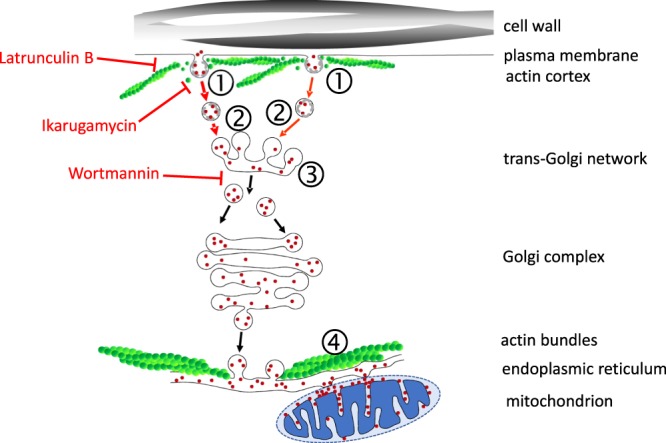
Working model for the cellular uptake of plant PeptoQ into tobacco BY-2 cells. The majority of the plant PeptoQ (red dots) enters via clathrin-dependent endocytosis, following interaction with a binding site (➀, & ➁, red arrows). The invagination of the plasma membrane requires a dynamic subpopulation of actin, which is subtending the membrane and can be eliminated by Latrunculin B. The endosomal vesicles (➁) converge at the trans-Golgi Network (TGN), which is the plant version of the early endosome (➂). From the TGN, the plant PeptoQ reaches the ER by retrograde transport via the Golgi complex (black arrows). The ER is structured by stable cables of actin that are also responsible for the movement of mitochondria. The membrane passage of the plant PeptoQ is proposed to occur at the mitochondria-associated ER membranes (➃). Through the porins of the outer mitochondrial membrane, the plant PeptoQ readily enters the intermembrane space of the mitochondrion. Due to the negative charge of the cytoplasmic face of the inner mitochondrial membrane, the positively charged plant PeptoQ is attracted and attached to this membrane. Consequently, it is removed from the outer face of the ER membrane and accumulated in the mitochondrion to levels that are much higher than those seen in the ER lumen. Note that the model is simplified and focussed on the route of uptake, aspects of membrane trafficking that are not relevant here (such as the multivesicular body or the vacuole, microtubules) have been deliberately omitted for the sake of clarity.

For the uptake of the plant PeptoQ, we arrive at a working model that describes a third scenario (Fig. 10): The plant PeptoQ is taken up by clathrin-dependent endocytosis depending on dynamic actin filaments. Then, it reaches the endoplasmic reticulum (ER) by retrograde transport passing early endosomes, trans-Golgi network, and the dictyosome^37–39^, from there, it can reach the ER by retrograde membrane flow (reviewed in^40,41^) and arrives at its final destination, the mitochondrial intermembrane space (presumably) at the contact sites between ER and mitochondria^42^. It is this final step, where the peptoid has to pass through a membrane-this membrane is neither the plasma membrane, nor the endosomal membrane, but the membrane of the ER. In the following section, we discuss the evidence leading to this working model.

### The uptake of the plant PeptoQ is saturable

Membrane passage of a cell-penetrating peptide might involve interaction with a binding site, in which case, uptake should be saturable. Alternatively, the membrane passage could proceed at any point of the membrane, independently of a particular binding site, as it seems to be the case for the Tat and Tat_2_ cell-penetrating peptides, where the entry into Triticale mesophyll protoplasts never became saturated^6^. The uptake of the plant PeptoQ is clearly saturable (Fig. 3). It seems that a first binding site has to be occupied, before the plant PeptoQ becomes available for the passage from the ER into the mitochondrion. This first binding site might reside on the plasma membrane and defines, how much peptoid can enter into clathrin-dependent endocytosis (Fig. 10, ➀&➁). The second bottleneck is the membrane passage itself, which likely occurs at the ER-mitochondrial contact points (Fig. 10, ➃). The size-exclusion of the porins in the outer mitochondrial membrane would be large enough to allow for back-diffusion from the intermembrane space to the cytoplasm but might be sequestered at the cytoplasmic face of the inner membrane, for instance, close to complex III^43^ from where it might even enter the matrix: since sodium ions can penetrate into the matrix through a proton-sodium antiporter system, leading to formation of a salt-induced permeability pore and loss of membrane tightness^44,45^. The reticulate patterns seen during early stages of uptake of a saturating peak with plant PeptoQ (Fig. 2) can be explained by this two-step model-this ER pool of labelled peptoid should become manifest as intermediary state at early time points, when the second step (the passage through the ER membrane) becomes limiting. This is because of the fact that plant PeptoQ uptake is time dependent where the first step (endocytotic uptake into the ER) is faster than the second step (ER to mitochondria). Thus, when high concentrations of plant PeptoQ administered the peptoid first accumulates in the ER and moves more slowly to the mitochondria. This biphasic behaviour might stem from the rapid depletion of the endocytotic machinery (such as clathrin which has to be recycled), irrespective of the continuous presence of the plant PeptoQ in the process. Thus, for high concentrations, the ER localization can be observed as intermediate step. When, instead, a lower concentration of peptoid is applied, this exhaustion of the endocytotic machinery does not occur and the intermediate pool located in the ER is observed more persistently also at later time points. Also, this implication of the hypothesis is supported by our data (Fig. 3 white arrowhead). As further implication this model predicts that a pulse-chase experiment should not produce the second “wave” of uptake seen for continuous incubation with saturating (2 µM) concentrations of the plant PeptoQ (Fig. 2). Also, this implication of the model could be experimentally confirmed (Fig. 2C).

The molecular nature of the two binding sites is not known at this stage, nor is the involvement of temperature-sensitive events, nor the potential mechanisms regulating membrane passage supposedly taking place at the ER-mitochondrial contact points. From structure-function relationships (reviewed in^46^), positively charged side groups have been determined as central factor for efficient uptake, whereby arginine and guanidine residues were more effective than lysines. In case of the plant PeptoQ, the positive charges will not only promote membrane passage, but may also be key factors for the strong partitioning to the mitochondria because the cytosolic face of the inner membrane (where the “natural” ubiquinone, mimicked by this peptoid, is located as well) is negatively charged due to the proton gradient across this membrane.

### Plant PeptoQ enters the cell via clathrin-dependent endocytosis

To understand, whether the plant PeptoQ undergoes a membrane passage at the plasma membrane, or whether it is first taken up through endocytosis, we have used two inhibitors of endocytosis, Wortmannin and Ikarugamycin (Fig. 5). While both inhibitors suppressed the uptake into mitochondria, the observed patterns were significantly different: After treatment with Wortmannin, only few residual signals could be seen at the cross walls, while with Ikarugamycin, the residual signals were significantly more abundant and also lined the lateral walls (Fig. 5). Unfortunately, the mode of action of the two inhibitors is far from clear, even for mammalian cells, but the published record supports a scenario, where Ikarugamycin blocks internalization of membrane receptors, while leaving intracellular trafficking untouched^47,48^. Wortmannin blocks phosphatidyl-inositol 3-kinase. Phosphorylated phosphatidyl inositol 3 interacts with the proteins that build up the retromer. Wortmannin, therefore, seems to interfere with a step of clathrin-dependent endocytosis downstream of the early endosome^30^. Consistent with this scenario, Wortmannin was found, for root cells of *Arabidopsis thaliana*, to cause rapid fusion of multivesicular bodies, a plant-specific precursor of vacuoles^49^. The plant PeptoQ is taken up by endocytosis, and this endocytosis is clathrin-dependent. To what extent clathrin-independent endocytosis contributes to uptake, warrants future investigations. By using a strategy, where the plasma-membrane located auxin-influx carrier AUX1 was expressed in fusion with yellow fluorescent protein (YFP) under an estradiol-inducible promoter, we were able to see the plant PeptoQ small vesicles close to the plasma membrane that presumably represented early endosomes (Fig. 6), and were accompanied by larger vesicles deeper in the cytoplasm that might be late endosomes supporting the conclusion from the experiments with endocytotic inhibitors. Unlike the situation in animals, endocytosis is completely independent of integrins (that have been lost early in plant evolution). Instead, cargoes are recognized through conformational motifs, linear amino acid motifs, and posttranslational modifications^50^.

### Membrane-associated actin is needed for endocytic uptake, microtubules are dispensable

Mitochondria were seen to be aligned with actin cables, and pretreatment with Latrunculin B eliminated uptake as efficiently as pretreatment with the endocytotic inhibitors, leaving only punctate signals lining the lateral cell membrane (Fig. 7). Also, some of the peptoid seemed to be contiguous with the actin cables, which does not necessarily mean that the signal is cytoplasmic, since most endosomal vesicles are smaller than the diffraction limit. These observations lead to the question of whether the tethering of ER and mitochondria by actin cables is required for mitochondrial targeting: (i) The role of actin cables for ER structure and movement of the Golgi complex in plant cells has been addressed in classical work^51^. (ii) The close link between mitochondria and ER structure has been shown by live imaging^37^ where there are mitochondria-associated membranes (MAMs) at mitochondria-ER contacts (MERCs) where the ER and the outer mitochondrial membranes (OMM) are about 20 nm apart. These contact sites between MAMs and mitochondria are responsible for the exchange of components between ER and mitochondria^52^. (iii) Plant mitochondria move along actin cables^53^. However, if Latrunculin B acts by interrupting the actin-dependent association of ER and mitochondria, the signal would be expected to remain trapped in the ER. This is not observed-the signal fails to enter the cell almost completely; only occasionally fluorescent foci were seen at the nuclei that had been displaced to the cross wall (Fig. 7) in consequence of the breakdown of the perinuclear actin cage that usually tethers the nucleus^54^. Our data indicate a different scenario linked to a different population of actin: Highly dynamic actin filaments subtend the cell membrane and participate in sensing membrane integrity^15^. Their proximity to the membrane is so close that it is possible to image fluorescently labelled actin filaments by Total Internal Reflection Fluorescence Microscopy in tobacco protoplasts^55^, indicative of a distance that must be well below 50 nm. We propose that Latrunculin B eliminates this membrane-associated, highly dynamic (and therefore also highly sensitive) population of actin filaments, which will intercept the formation of membrane invagination and, thus, block endocytotic uptake. In fact, the role of actin filaments for endocytotic uptake in mammalian and yeast cells is well established (for review, see^56^), and has also been observed in plants, where the internalization of the auxin-transport facilitator PIN1 was blocked by cytochalasin D^57^. Since the anchoring of mitochondria has been linked with microtubules^53^, and since the microtubule +TIP protein CLASP was found to participate in endocytotic cycling of PIN2^58^, a role of microtubules for peptoid uptake is plausible. In fact, the uptake of polyguanidine peptoids (mimicking a polyarginine peptide) into tobacco BY-2 cells was inhibited by Oryzalin, although only partially. Although we could see, using a fluorescent tubulin marker line, that some mitochondria co-localized with microtubules (Fig. 8), the orientation of the mitochondria often deviated from the direction of the tethering microtubules indicative of a different lattice (actin cables) intersecting with the microtubules. Most importantly, when we eliminated microtubules using Oryzalin, the plant PeptoQ was still taken up in the same manner as in the controls, where microtubules were intact. These findings are in line with the literature report, where actin is responsible for most of the intracellular movements of organelles including ER, endosomes, Golgi stacks, vesicles, and mitochondria^59^. Thus, in contrast to actin filaments, microtubules are dispensable for uptake and mitochondrial targeting of the plant PeptoQ.

### Plant PeptoQ efficiently alleviates stress-induced programmed cell death

The peptoid used in the current study represents a novel approach to deliver functional cargoes to the mitochondria. The target of this approach is to improve the mitochondrial redox homeostasis under salinity. Specifically, the ionic component of salinity stress impairs the electron transport chain (ETC) complexes^45,60–62^ such as complex I (NADH dehydrogenase) and complex II (succinate dehydrogenase)^63^. The impairment of ETC complexes, and the inhibition of the respiratory rate, lead to the overreduction of ETC and elevated leakage of electrons onto O_2;_ eventually, resulting in increased production rate of superoxide and mitochondrial oxidative stress^45,64^. In plants, an alternative oxidase (localized at the matrix face of the inner membrane) provides a bypass and becomes dominating during salt stress, because it remains unaffected, as long as no permeability pore is produced^44,45^.

To what extent the peptoid acts on the mitochondrial electron transport, remains to be elucidated. A straightforward approach would be to compare respiratory activity of tobacco cells under salt stress with and without peptoid. In plant cells, this is not as conclusive, because the mitochondrial respiration is marked by abundant cell wall peroxidase that are even induced under salt stress^65^. We have therefore used, mitochondrial coverage as readout, which is an indicator of disturbed redox homeostasis^66^. The observed breakdown of this parameter under salt stress, and the mitigation of this breakdown by plant PeptoQ are consistent with the redox activity of this peptoid, which is currently followed up in more detail. From the structure, the quinone within the peptoid is identical to the “native” CoQ10 with exception of the isoprenoid side chain. It has been reported that the hydroxylated forms of CoQ10 (CoQs in general) harbour very strong antioxidant and superoxide scavenging properties^25–27^. The increased electronic density that emanates from the OH groups accounts for the strong antioxidative properties of the hydroxyl derivatives of CoQs^26^. The hydroxylation of CoQs *in vivo* would take place by the action of hydroxylating enzymes or monooxygenases^25,67,68^. Thus, since the side chain does not have any influence on the redox state of CoQ10 (CoQs), plant PeptoQ, will have similar redox property and antioxidative potential like the hydroxylated CoQs. In support of these *in vitro* data, we saw a mitigation (and even inversion) of salt-induced accumulation of superoxide (Suppl. Fig. S4), as well as a suppression of mitochondrial fragmentation under salt stress (Fig. S3). Both findings indicate the entrance of PeptoQ into the matrix during salt induced formation of the mitochondrial permeability pore. Thus, we can confirm the antioxidative activity of plant PeptoQ also *in vivo*.

Of course, alternative strategies to peptoid delivery are possible and have been already employed in plants^69,70^ fusions of mitochondrial and plastid transit peptides with a charged domain binding DNA have been used to engineer the genomes of both organelles. By adding the cell-penetrating BP100, the efficiency of delivery could be increased^66^ indicating that passage through the plasma membrane was limiting for these constructs. While the use of peptides as carrier is convenient and versatile, the need of the transit peptides to travel through the cytoplasm might also have certain limitations: Even a transient perturbation of membrane integrity can activate a sensory circuit composed of the membrane-located nicotinamide adenine dinucleotide phosphate (NADPH) oxidase RboH and membrane-associated actin which acts as trigger for programmed cell death^15^. By hijacking the endosome-ER-mitochondria pathway, this problem can be circumvented. In fact, even for high concentrations of peptoid, we could not detect any indication of elevated mortality (Suppl. Fig. S1), which is in stark contrast to the behaviour served with the cell-penetrating peptide BP100. For the purpose of organelle transformation, where most cells will be eliminated anyway by subsequent selection, this aspect may not be so relevant. However, for where the response to altered mitochondrial physiology is to be studied, this aspect is crucial. Here, the peptoid strategy brings significant payoff that makes up for the somewhat more cumbersome synthesis (although modular strategies of peptoid synthesis have strongly reduced this challenge). A further benefit for long-term studies, is the inaccessibility of the peptoid bond to protease activity.

## Conclusion and Perspectives

### The plant PeptoQ as tool to dissect spatial signatures of oxidative stress

Subcellular targeting to mitochondria has attracted considerable attention for biomedical applications, since mitochondria are not only crucial for energy acquisition, but also regulate numerous physiological and pathological processes (reviewed in^71^). For plant cells as well, mitochondria have been intensively studied as central factors of oxidative balance and for the initiation of programmed cell death, for instance in the context of salt-stress (recently reviewed in^72^). Sodium ions can enter the plasma membrane through non-selective cation channels and then activate oxidative signaling that either results in cellular adaptation or in salt-induced necrosis where the target cell sequesters sodium ions from its neighbours and removing it from the system, for instance, by abscission of tissue to protect the young meristem. The response depends not only on the molecular nature of early signals, but mainly on their temporal and spatial pattern (reviewed in^73^). Reactive oxygen species can convey a completely different signal, depending on the site of their occurrence (so called spatial signature). To experimentally address such a signature model requires the control of oxidative balance differentially in different subcellular compartments. For instance, the sodium ions entering the cytoplasm, can pass through porins in the outer mitochondrial membrane^36^, will accumulate at the cytoplasmic face of the inner mitochondrial membrane (reviewed in^36,45,63,72^) and trigger the accumulation of superoxide (reviewed in^74^). Superoxide production is also triggered at the plasma membrane by NADPH oxidase and seems to act as a signal, which has also been demonstrated for tobacco BY-2 cells^75^. It will be interesting to examine, if the plant PeptoQ gets integrated into the electron transport chain of the inner membrane and affects oxidative phosphorylation there. This raises the question, whether the peptoid can pass the inner membrane. This may not be the case, because the lipid composition of the inner membrane, differs sharply from the other plant membranes, because it is prokaryotic type, for instance, with high contents of cardiolipins. Under control conditions, when the inner membrane is tight, we do not see any effect of plant PeptoQ. Access to the matrix might become relevant, however, when under salt stress a permeability pore is formed^76^ such that the peptoid can enter. We already know that the plant PeptoQ does not induce any detectable toxicity by itself but is able to mitigate salt-stress dependent programmed cell death. This effect is independent of the conjugated rhodamine. Although salt stress is a serious problem in global agriculture with sharply rising impact, application of this peptoid in agriculture would not be economically feasible, and this is also not what we are planning to develop. We rather want to use this new tool to dissect the functional role of plasma membrane and mitochondria as important cellular sites of superoxide accumulation. In this context, synthetic plant PeptoQ variants with a more negative redox potential are of interest, because they may allow for even more efficient elimination of superoxide and hydrogen peroxide. We are currently completing a study, where we follow this up in more detail and find that plant PeptoQ can effectively reduce salt stress-induced accumulation of ROS (mainly superoxide), mitigate lipid peroxidation, and activate the mitochondrial superoxide dismutase. Our current strategy to tailor the functional cargo, can be accompanied by strategies to tailor the molecular vehicle: We have already launched a combinatorial approach, where different peptoids are synthesized from modular building blocks to get insight into the structural features that can be used to optimise the different steps of uptake. The goal of these endeavours is to obtain a tool to shape oxidative balance in mitochondria as a lever to control adaptive gene expression, for instance, to promote salt tolerance in plant cells. It would be the genetic components identified by this approach that would then be the starting point for agricultural application, for instance, as targets in marker-assisted breeding for salt tolerance.

## Materials and Methods

### Synthesis and labelling of the plant PeptoQ 3

The plant PeptoQ 3, is the alias for a rhodamine B labeled cell penetrating peptoid, covalently connected to the ubiquinone analogue 6-(10-azidoalkyl)-benzoquinone (Fig. 1). containing an omega azidodecyl residue at position 6 instead of an isoprenyl moiety of CoQ10 **1**. As seen, the Quinone within the peptoid is identical as the “native” CoQ10 with exception of the isoprenoid side chain. Given that the redox properties of this type of benzoquinones are determined by the ketone groups at position 1 and 4, where the side chain has no impact on this property^28^. Thus, we are convinced that plant PeptoQ endowed with the same benzoquinone head as CoQ10, is a redox active molecule and has redox properties which are comparable with those of Coenzyme Q10. Moreover, since the hydroxyl derivatives of CoQ10 owns very strong antioxidative potential^25–27^ plant PeptoQ has also remarkable antioxidative and superoxide scavenging potential.

### Synthesis of 2,3-dimethoxy-5-methyl-(6-(10-bromodecyl)-1,4-benzoquinone (2)

6-(10-bromodecyl) ubiquinone was synthesized according to previous reports by^77,78^. In a first step, 11-bromoundecanoic peroxide was synthesized by heating 11-bromoundecanoic acid (4.00 g, 15.1 mmol) and SOCl_2_ (1.60 ml, 21.5 mmol) at 90 °C for 15 min. Excess of SOCl_2_ was removed by distillation under reduced pressure (15 mm Hg, 90 °C) and the residue (IR,1 1799 cm^−1^) was dissolved in diethyl ether (20 ml) and subsequently cooled to 0 °C on ice. Hydrogen peroxide (30%, 1.80 ml) was added followed by dropwise addition of pyridine (1.40 ml) over 45 min. Eventually, diethyl ether (10 ml) was added and stirred for 1 h at room temperature. The product was diluted with diethyl ether (150 ml), washed with H_2_O (2 × 70 ml), 1.20 M HCl (2 × 70 ml), H_2_O (70 ml), 0.50 M NaHCO_3_ (2 × 70 ml), and H_2_O (70 ml). After drying over MgSO_4,_ the solvent was removed at room temperature under reduced pressure, giving crude product as a white solid (2.89 g), and processed immediately. 6-(10-Bromodecyl)-ubiquinone (**2**) was synthesized by stirring the crude product (2.89 g, 10.3 mmol), 2,3-dimethoxy-5-methyl-1,4-benzoquinone (1.01 g, 6.00 mmol), and acetic acid (60 ml) for 20 h at 100 °C. After cooling to room temperature, the reaction was diluted with diethylether (600 ml), washed with H_2_O (2 × 400 ml). Evaporation of the solvent under reduced pressure yielded in a reddish solid (4.31 g). Column chromatography on silica gel, eluting with CH_2_Cl_2_, yielded in a red oil (682 mg, 1,70 mmol, 28.32%), which was not further purified. NMR (299.9 MHz) 3.99 (s, 6H, 2x-OC**H**_3_), 3.41 (t, *J* 5 6.8 Hz, 2H, –C**H**_2_–Br), 2.45 (t, *J* 5 7.7 Hz, 2H, ubquinone–C**H**_2_–), 2.02, (s, 3H, –C**H**_3_). 1.89 (quin, *J* 5 7.4 Hz, 2H, –C**H**_2_–CH_2_-Br), 1.42–1.28 (m, 14H, -(C**H**_2_)_7_-) ppm; ^13^C NMR (125.7 MHz)184.7 (**C**=O), 184.2 (**C**=O), 144.3 (2**C**, ring), 143.1 (ring), 138.7 (ring), 61.2 (2x-O**C**H_3_), 34.0 (–C**H**_2_–), 32.8 (–C**H**_2_–), 29.8 (–C**H**_2_–), 29.4 (2x–C**H**_2_–), 29.3 (–C**H**_2_–), 28.7 (23 –C**H**_2_–), 28.2 (–C**H**_2_–), 26.4 (–C**H**_2_–), 11.9(–**C**H_3_) ppm. MALDI-TOF, matrix: DHB, m/z (%): 401 [M]^+^.

### Solid-phase synthesis

Peptoid syntheses were performed on solid-phase following the fluorenylmethyloxycarbonyl (Fmoc)-strategy by using Boc-protecting groups for the side chains to facilitate the synthesis of the growing oligomer^79,80^. Rink-amide-resin was chosen as a solid support due to its stability at ambient conditions, the ease of the first coupling step, and good cleavage conditions. Furthermore, the reaction conditions were the same for attaching the first building block to the resin and the following coupling cycles. After removal of the Fmoc group, which protected the amino-functionalized resin (with 20% piperidine in dimethylformamide (DMF)), an activated Fmoc-protected monomer was coupled to the solid phase via a peptide bond. For the microwave-assisted reactions HOBt and DIC were used as coupling reagents. The Fmoc group was removed with piperidine-solution yielding the coupled monomer for the attachment of the next building block. All reaction procedures were succeeded by repetitive washing, ending with a solvent, in which the resin was swelled to expose its reactive sites to the next reagents. The cycles of coupling and deprotection were repeated until a peptoid of the desired length was obtained.

### Microwave-assisted synthesis of Boc-protected hexamer on a Rink amide linker (Procedure 1)

The resin (AM resin LL 100-200 mesh, 0.61 mmol/g, 218 µmol, 1.00 equiv) was covered with five times of its volume of dried DMF and swelled for 30 min. After deprotection of Fmoc (3 × 5 min with 3 mL of 20% piperidine in DMF) and thoroughly washing with DMF the acylation of the resin was done with 0.6 g bromoacetic acid (4.36 mmol, 20 equiv) and diisopropylcarbodiimide (DIC) (0.55 g, 4.36 mmol, 20 equiv) in DMF for 2 h (Suppl. Fig. S2). After the reaction the resin was washed 3 × 20 min with DMF. For the coupling with propargylamine the resin was incubated with a 1 M of propargylamine in DMF overnight. Eventually the resin was washed three times with DMF and the building block *N*-(6-*tert*-butoxycarbonylamino-hexyl)-*N*-(9*H*-fluoren-9-ylmethoxycarbonyl) acetic acid (653 µmol, 3.00 equiv), HOBt (653 µmol, 3.00 equiv) and DIC (653 µmol, 3.00 equiv) were dissolved in DMF biotech grade (6.50 mL) to obtain a 0.1 M solution related to the building block. The reaction solution was added to the resin and stirred for 30 min at 60 °C in a CEM microwave oven. The reaction solution was filtered, and the resin was treated a second time with freshly prepared reaction solution for 30 min at 60 °C in the microwave oven (double coupling) and subsequently incubated with 3 mL of 20% piperidine in DMF (3 × 5 min) to cleave the Fmoc group. The resin was not dried after the reactions. This reaction was repeated six times to obtain the resin bound hexamer as yellowish solid.

### Microwave-assisted rhodamine B-labeling of the peptoid (Procedure 2)

After the deprotection of Fmoc (3 × 5 min with 3 mL of 20% piperidine in DMF) and accurate washing with DMF. Rhodamine B (653 µmol, 3.00 equiv), HOBt^•^H_2_O (653 µmol, 3.00 equiv) and DIC (653 µmol, 3.00 equiv) were dissolved in DMF biotech grade (6.50 mL) to obtain a 0.1 M solution related to the dye. The reaction solution was added to the resin of procedure 1 and stirred for 30 min at 60 °C in a CEM microwave oven. The reaction solution was filtered, and the resin was treated a second time with freshly prepared reaction solution for 30 min at 60 °C in the microwave oven (double coupling). The resin was not dried after the reactions.

### 2,3-Dimethoxy-5-methyl-(6-(10-bromodecyl)-1,4-benzoquinone coupling (Procedure 3)

To couple the 2,3-dimethoxy-5-methyl-(6-(10-bromodecyl)-1,4-benzoquinone via a 1,3-dipolar cycloaddion (click reaction), the resin from procedure 2 was reacted according to^81,82^. In a one pot reaction with2,3-dimethoxy-5-methyl-(6-(10-bromodecyl)-1,4-benzoquinone (131 mg, 327 µmol, 1.50 equiv) and sodium azide (327 µmol, 1.50 equiv) in DMF at room temperature. To this solution, a CuSO_4_ solution (10 mol%) was added. Eventually, sodium ascorbate (30 mol%) and deionized water (5 vol%) was added. The temperature was then increased to 50 °C and the resin was agitated for 2 d. After agitating for 2 d the reaction mixture was removed and the resin was washed thoroughly with DMF.

### Microwave-assisted Boc-deprotection on solid phase (Procedure 4)

For the deprotection of the Boc-functionalized amino groups, the resin of procedure 3 (218 µmol, 1.00 equiv) was covered with 3 mL of a 3 M hydrochloric acid in DMF solution and stirred for 100 min at 120 °C in a CEM microwave oven. The resin was washed with DMF.

### Microwave-assisted guanidinylation on solid phase (Procedure 5)

After the Boc deprotection of the side-chain amines the resin from procedure 4 (218 µmol, 1.00 equiv) was covered with a solution of 1*H*-pyrazol-1-carboxamidine (1.09 mmol, 20.0 equiv), DIPEA (2.18 mmol, 40.0 equiv) and 3.20 mL of DMF. The reaction was performed in a CEM-microwave (120 min reaction time, 60 °C). The resin was washed with DMF.

### Cleavage and isolation (Procedure 6)

To cleave the peptoid from solid support, a solution of 2.00 mL TFA in dichloromethane (95:5 (v/v) was added to the resin of procedure 5 and shaken for 2 h. The resin was rinsed two times with 0.5 mL methanol. Water was added to the solution and the sample was frozen and lyophilized and purified by HPLC.

### Plant PeptoQs

Using general procedures 1, (2), 3 - 6 the product was obtained as a red solid. HPLC purification and lyophilisation yielded 15.39 mg (7.36 µmol, 3.38% over 20 steps) of Rhodamine-labeled **plant PeptoQ 3**. with a purity of >98%. MS (MALDI-TOF, matrix: DHB, m/z (%): 1993 [M]^+^ and 18.74 mg (11.3 µmol, 5.16% over 19 steps) of non-labeled plant PeptoQ 4 with a purity of 93%. MS (MALDI-TOF, matrix: DHB, m/z (%): 1567 [M]^+^_._

### HPLC purification

Preparative high performance liquid chromatography (HPLC) was performed on an *Äkta* Purifier 100 UPC equipped with a pump P-900, monitor UV-900 and UPC-900, Valve INV-907, Mixer M-925 and Valve PV-908 employing a reverse phase C18 semi-preparative column (*Macherey Nagel* VP 250/10 Nucleodur 100-5 C18ec), flow rate 1.5 mL/min, solvent A: 0.1% trifluoroacetic acid (TFA) in water, B: methanol and *Agilent* 1200 Series equipped with a diode array detector employing a reverse phase C8 semi-preparative column (Zorbax 300SB-C8 (*Agilent*), 5 µm, 9.4 mm × 250 mm), flow rate 1 mL/min, A: 0.1% TFA in water, B: 0.1% TFA in acetonitrile for purification assessment. Analytical high-performance liquid chromatography (HPLC) was performed on an *Agilent* 1100 Series equipped with a diode array detector employing a reverse phase C18 analytical column (PerfectSil Target (*MZ-Analytik*), 5 μm, 4.0 mm × 250 mm), flow rate 1 mL/min, A: 0.1% TFA in water, B: 0.1% TFA in acetonitrile for purification assessment. Analytical and preparative high-performance liquid chromatography (HPLC) was performed on a chromatographic system from Jasco (Tokyo, Japan) equipped with diode-array detector. Reverse phase C18 analytical (4.6 × 250 mm, 5 µm) or semi-preparative (10 × 250 mm, 10 µm) columns from Grace (Grace, Deerfield, IL) were employed for purity assessment and purification respectively. For chromatographic separation of the peptoids, focused gradients were run from 48% to 56% at a constant temperature of 40 °C. Solvent A: 0.1% trifluoroacetic acid (TFA); B: 90% acetonitrile in 0.1% TFA. The separation of the peptoids was monitored with UV-detection in the range of 200–650 nm and ultraviolet (UV) spectra along with matrix-assisted laser desorption/ionization-time of flight (MALDI-TOF)-mass spectrometry (MS) to identify the product peaks. Manually collected fractions of the semi-preparative runs were freeze-dried and immediately used in the biological assays. Prior to lyophilization, fraction aliquots were directly re-injected onto the analytical column to quantify purity, which was determined by integration of the respective single peak area from the chromatograms at 218 nm.

### Cell lines and cell cultivation

Suspension cell lines of tobacco (*Nicotiana tabacum* L. cv Bright Yellow-2^83^) were grown in liquid medium containing 4.3 g/L Murashige and Skoog (MS) salts (Duchefa Biochemie, The Netherlands), 30 g.L^−1^ sucrose, 200 mg.L^−1^ KH_2_PO_4_, 100 mg.L^−1^ (myo)-inositol, 1 mg.L^−1^ thiamine, and 0.2 mg.L^−1^ 2,4-D, pH 5.8. At weekly intervals, 1.5 mL of stationary cells were inoculated into a 30 mL Erlenmeyer flask with fresh medium and shaken in the dark at 26 °C on a KS260 basic orbital shaker (IKA Labortechnik, Germany) at 150 rpm. Stock BY-2 calli were maintained on media solidified with agar [0.8% (w/v)] and subcultured monthly. In addition to the non-transformed wild type, transgenic cell lines were used in this study and were supplemented with the respective selective agent: To visualize actin filaments, the marker line GF11^84^ expressing the actin-binding domain of plant fimbrin was used and cultivated in presence of hygromycin (30 mg. L^−1^). To observe microtubules, the transgenic line TuB6, expressing the beta-tubulin AtTUB6 from *Arabidopsis thaliana* fused to GFP driven by the Cauliflower mosaic virus (CaMV) 35S promotor^55^ was employed and supplemented with kanamycin (50 mg. L^−1^). In order to observe the behaviour of the plant PeptoQ in relation to early endosomes, a line expressing the auxin influx carrier AUX1 in fusion with YFP from *Arabidopsis thaliana* under control of an estradiol-inducible promoter^35^ was cultivated in presence of hygromycin (40 mg. L^−1^) and induced by beta-estradiol (1 µM) for 24 h at day 2 after subcultivation. If not stated otherwise, all treatments were conducted with tobacco BY-2 cells collected at the peak of the proliferation phase (day 3 after subcultivation).

### Treatment with rhodamine-labelled plant PeptoQ, inhibitor treatments, and fluorescent dyes

To record the dose-response relation (Fig. 3), non-transformed tobacco BY-2 cells were collected at the peak of proliferation (day 3 after subcultivation) and incubated with 0.5–6 µM of plant PeptoQ for 2 h, and then washed three times before observation. To measure the time course of uptake (Fig. 2), the cells were treated with 2 µM plant PeptoQ for 10, 30, 60, 90, 120, 180, 240, 300 and 360 min. During this incubation time, the peptoid was present throughout. To probe for the role of different cellular component or event for uptake, cells were pretreated with different inhibitors before adding 2 µM of the rhodamine-labelled plant PeptoQ and analyzing the result after 2 h of uptake. To verify the effect of the inhibitor, appropriate fluorescently labelled marker lines were used, alternatively fluorescent dyes specific for the respective process. To probe for the role of actin filaments, the actin marker line GF11 was pretreated for 1 h with 10 µM of Latrunculin B (Lat B, Sigma–Aldrich, Taufkirchen, Germany), while the role of microtubules was tested by pretreatment with 10 µM of Oryzalin over 1 h (Sigma–Aldrich, Taufkirchen, Germany) in the microtubule-marker line TuB6. Both inhibitors were diluted from a stock solution in dimethylsulfoxide (DMSO) with the culture medium to get the final working concentration. Clathrin-dependent endocytosis was inhibited by 33 µM Wortmannin (Sigma–Aldrich, Taufkirchen, Germany), or by 10 µM Ikarugamycin (IKA, Sigma–Aldrich, Taufkirchen, Germany), both incubated for 30 min before adding the plant PeptoQ. Here, the polystyryl dye FM4-64 (2 µM, 5 min incubation) was used as readout for the state of endocytosis. To verify the mitochondrial localization, cells were incubated over 2 h with 2 µM of rhodamine-labelled plant PeptoQ before adding the specific dye MitoTracker Green (Molecular Probes) in 0.1% (v/v) 5 min prior to microscopic observation. Superoxide content in response to salt stress in presence or absence of plant PeptoQ was quantified based on a nitro blue tetrazolium (NBT) based approach according to^85^. To test for potential toxicity (2, 4, 8, 16, 25, 40 and 50 µM of plant PeptoQ) on the one hand, and a potential functionality for the mitigation of salt-induced programmed cell death on the other, mortality was followed over time using the Evans Blue dye exclusion test^86^ after addition of 75 mM NaCl at the time of subcultivation, either with or without supplementation with 2 µM of plan PeptoQ. Mortality was followed on a daily base till day 4 after start of the stress treatment. Mortality was determined as relative proportion of cells that after thorough washing had retained the blue dye. Each data point represents average and standard error from at least 1000 cells counted and collected from at least three independent experimental series.

### Microscopy

Except in the case of MitoTracker staining, cells were washed thoroughly in culture medium and then immediately visualized under the microscope. Aliquots of 30 µl were mounted on a microscope slide, covered with a cover slip, and viewed under an AxioObserver Z1 microscope (Zeiss, Jena, Germany) that was equipped with a spinning-disc device (YOKOGAWA CSU-X1 5000) and a cooled digital charge-coupled device (CCD) camera (AxioCam MRm). The signal from GFP and MitoTracker Green was activated using the 488-nm line of an Ar-Kr laser (Zeiss), while the signal from rhodamine and FM4-64 was activated through the 561-nm line of the same laser. Images were operated via the Zen 2012 (Blue edition) software platform. Mortality scores were conducted by means of an Axioskop microscope (Zeiss, Jena, Germany), equipped with a 32x long distance objective (Zeiss Neofluar, Jena, Germany), and a digital CCD camera (AxioCam MRm).

### Quantitative image analysis and modelling of uptake

Uptake was quantified as described in^15^ making use of the Image J software (http://rsb.info.nih.gov/ij/). It is important that image acquisition is standardized with respect to laser power and exposure time, which requires that automatic optimization tools are inactivated during imaging. Fluorescence intensity in confocal sections in the cell center, was averaged over the interior of the cell and corrected against the background of a calibration area chosen from the environment of the measured cell. The regions of interest were selected using the freehand selection tool of Image J.

To quantify the association of the plant PeptoQ signal with other fluorescent markers labelling different compartments, the channels of the merged image were split, and then the individual pixel intensities of the red channel (recording the plant PeptoQ signal) were subtracted from the green channel (recording the respective fluorescent marker). Mean pixel intensity of the resulting differential image was then divided by the mean pixel intensity of the green channel for normalisation. The resulting ratio would be 0% in case the two signals matched completely, while it would be 100% in case the two signals were completely mutually conclusive.

## Supplementary information

Supplementary Figures with legends

## Data Availability

All data generated and analysed in the current study are available from the corresponding author on reasonable request.
