# Author Correction: A Peptoid Delivers CoQ-derivative to Plant Mitochondria via Endocytosis

**DOI:** 10.1038/s41598-019-54996-0

**Published:** 2019-12-06

**Authors:** Kinfemichael Geressu Asfaw, Qiong Liu, Jan Maisch, Stephan W. Münch, Ilona Wehl, Stefan Bräse, Ivan Bogeski, Ute Schepers, Peter Nick

**Affiliations:** 1 Molecular Cell Biology, Botanical Institute, Karlsruhe Institute of Technology (KIT), Fritz-Haber-Weg 4, D-76131 Karlsruhe, Germany; 2 Institute of Organic Chemistry, Karlsruhe Institute of Technology (KIT), Fritz-Haber-Weg 6, D-76131 Karlsruhe, Germany; 3 Molecular Physiology, Institute of Cardiovascular Physiology, University Medical Center, Georg-August-University, 37073 Göttingen, Germany; 4 Institute of Toxicology and Genetics (ITG), Karlsruhe Institute of Technology (KIT), Hermann von Helmholtz Platz 1, D-76344 Eggenstein-Leopoldshafen, Germany; 5 Institute of Functional Interfaces (IFG), Karlsruhe Institute of Technology (KIT), Hermann von Helmholtz Platz 1, 76344 Eggenstein-Leopoldshafen, Germany

Correction to: *Scientific Reports* 10.1038/s41598-019-46182-z, published online 08 July 2019

The original version of this Article omitted an affiliation for Ilona Wehl and Stefan Bräse. The correct affiliations for Ilona Wehl and Stefan Bräse are listed below:

Institute of Organic Chemistry, Karlsruhe Institute of Technology (KIT), Fritz-Haber-Weg 6, D-76131 Karlsruhe, Germany.

Institute of Toxicology and Genetics (ITG), Karlsruhe Institute of Technology (KIT), Hermann von Helmholtz Platz 1 D-76344 Eggenstein-Leopoldshafen, Germany.

Additionally, in the Supplementary Information file originally published with this Article the treatment in Supplementary Figure S1 was incorrectly listed as CoQ Peptoid.

These errors have now been corrected in the HTML and PDF versions of this Article, and in the accompanying Supplemental Material.

