# Supplementary material for: A Peptoid Delivers CoQ-derivative to Plant Mitochondria via Endocytosis
Source: Sci Rep. 2019 Jul 8;9:9839. doi: 10.1038/s41598-019-46182-z (PMC6614412; doi:10.1038/s41598-019-46182-z)
Supplement: Supplementary file 1 — Supplementary Figures with legends [file 41598_2019_46182_MOESM1_ESM.pdf]

# A Peptoid Delivers CoQ-derivative to Plant Mitochondria via Endocytosis

Kinfemichael Geressu Asfaw[1], Qiong Liu[1], Jan Maisch[1], Stephan W. Münch[2], Ilona Wehl[2,4], Stefan Bräse[2,4], Ivan Bogeski[3], Ute Schepers[2,5], Peter Nick[1]

[1]Molecular Cell Biology, Botanical Institute, Karlsruhe Institute of Technology (KIT), Fritz-Haber-Weg 4, D-76131 Karlsruhe, Germany

[2]Institute of Organic Chemistry, Karlsruhe Institute of Technology (KIT), Fritz-Haber-Weg 6, D-76131 Karlsruhe, Germany

[3]Molecular Physiology, Institute of Cardiovascular Physiology, University Medical Center, Georg-August-University, 37073 Göttingen, Germany

[4]Institute of Toxicology and Genetics (ITG), Karlsruhe Institute of Technology (KIT), Hermann von Helmholtz Platz 1 D-76344 Eggenstein-Leopoldshafen, Germany

[5]Institute of Functional Interfaces (IFG), Karlsruhe Institute of Technology (KIT), Hermann von Helmholtz Platz 1 76344 Eggenstein-Leopoldshafen, Germany

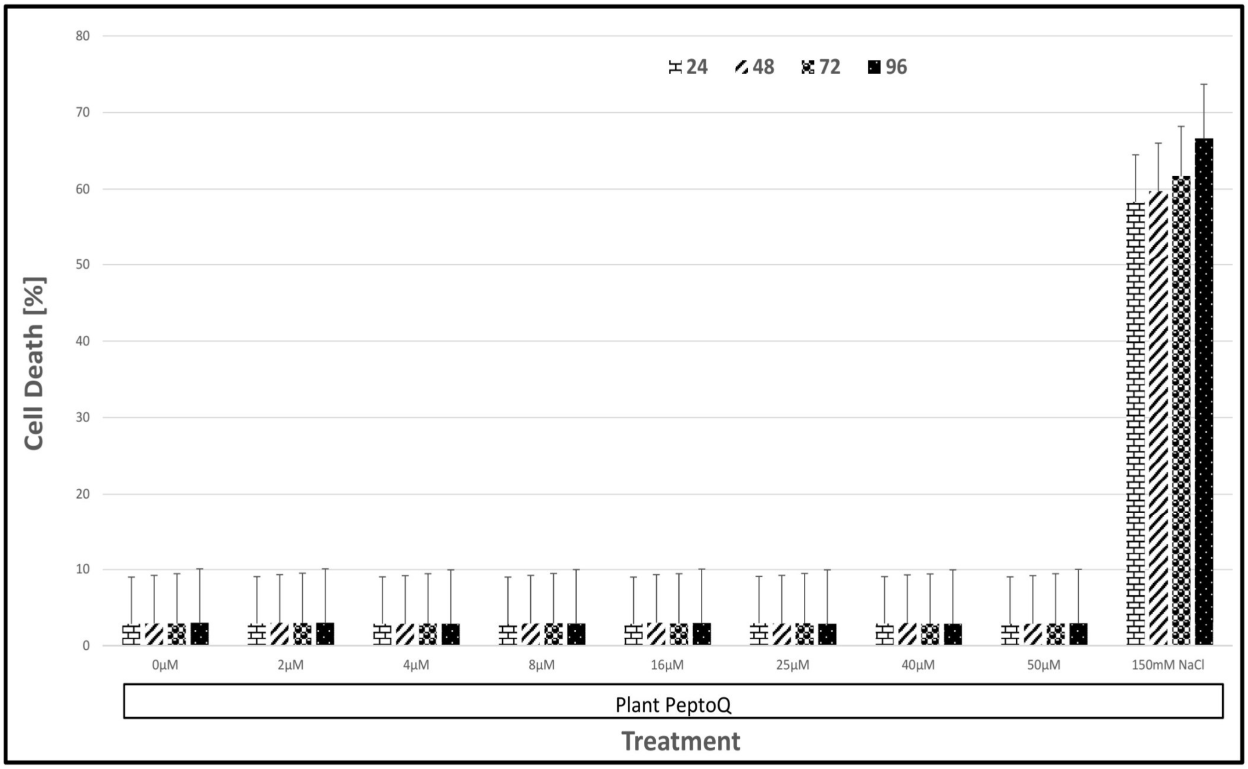


**Supplementary Figure S1**: Effect of plant PeptoQ in different concentrations (from 0-50 µM) and salinity stress upon viability of tobacco BY-2 cells scored at different time points up to 4 days. Data represent mean and standard errors from three different biological replicates, representing a sample population of 1000 cells per replicate.

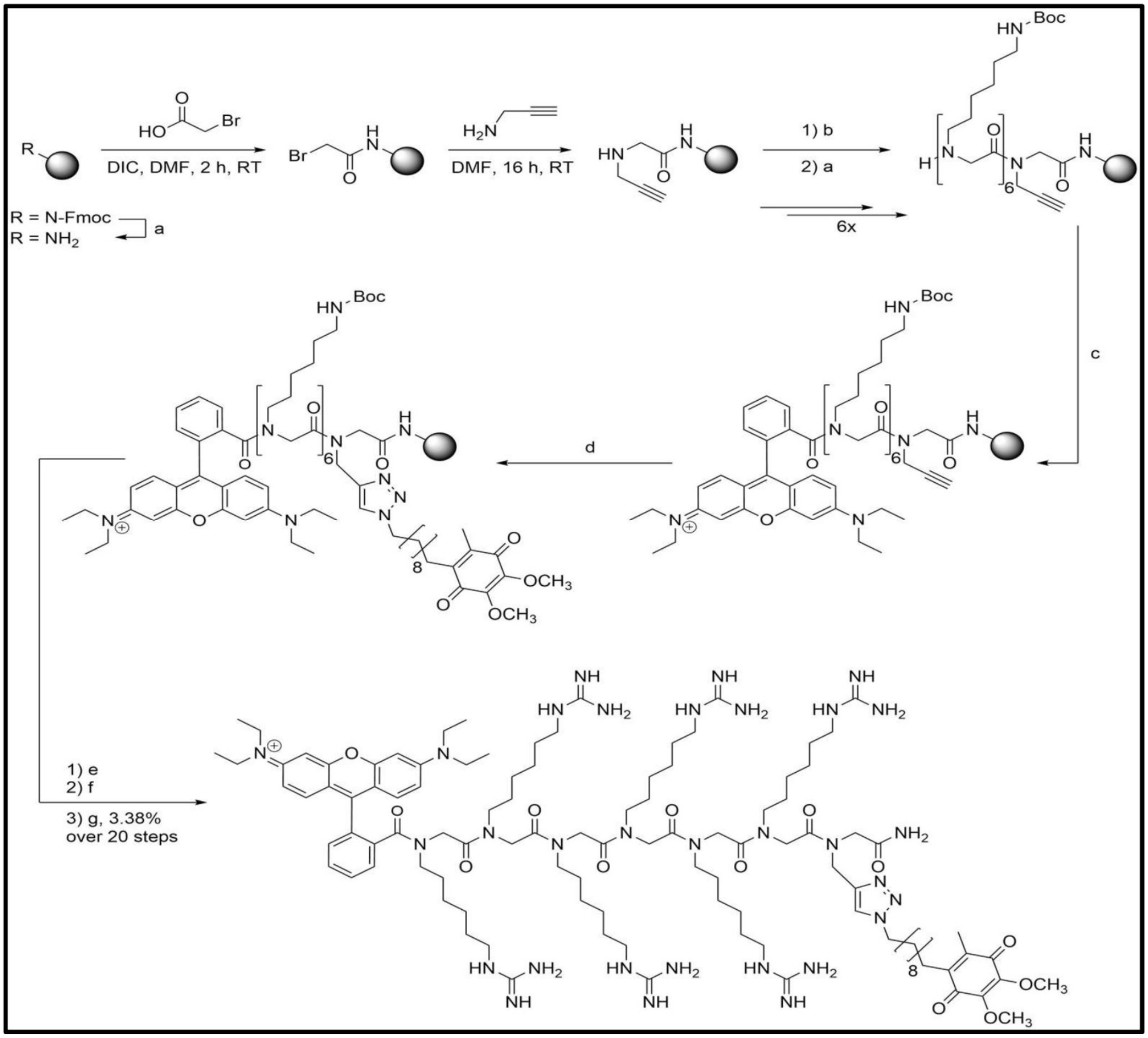


**Supplementary Figure S2:** Synthesis scheme of the plant PeptoQ 3. Reaction conditions: a) 20% piperidine in DMF, 3 × 5 min, RT; b) *N*-(6-*tert*butoxycarbonylamino-hexyl)-*N*-(9*H*-fluoren-9-ylmethoxycarbonyl)acetic acid, DIC, HOBt, DMF, 30 min, 60 °C microwave; c) Rhodamine B, DIC, HOBt, DMF, 30 min, 60 °C microwave; d) 6-(10-bromodecyl)-2,3-dimethoxy-5-methylcyclohexa-2,5-diene-1,4-dione, NaN3, CuSO4, sodium ascorbate, DMF, water, 2 d, RT; e) 3 M HCl in DMF, 100 min, 120 °C microwave; f) 1*H*-pyrazol-1-carboxamidine, DIPEA, DMF, 120 min, 60 °C microwave; g) TFA/dichloromethane (95:5 (v/v)), 2 h, RT; 3.38% yield over 20 steps.

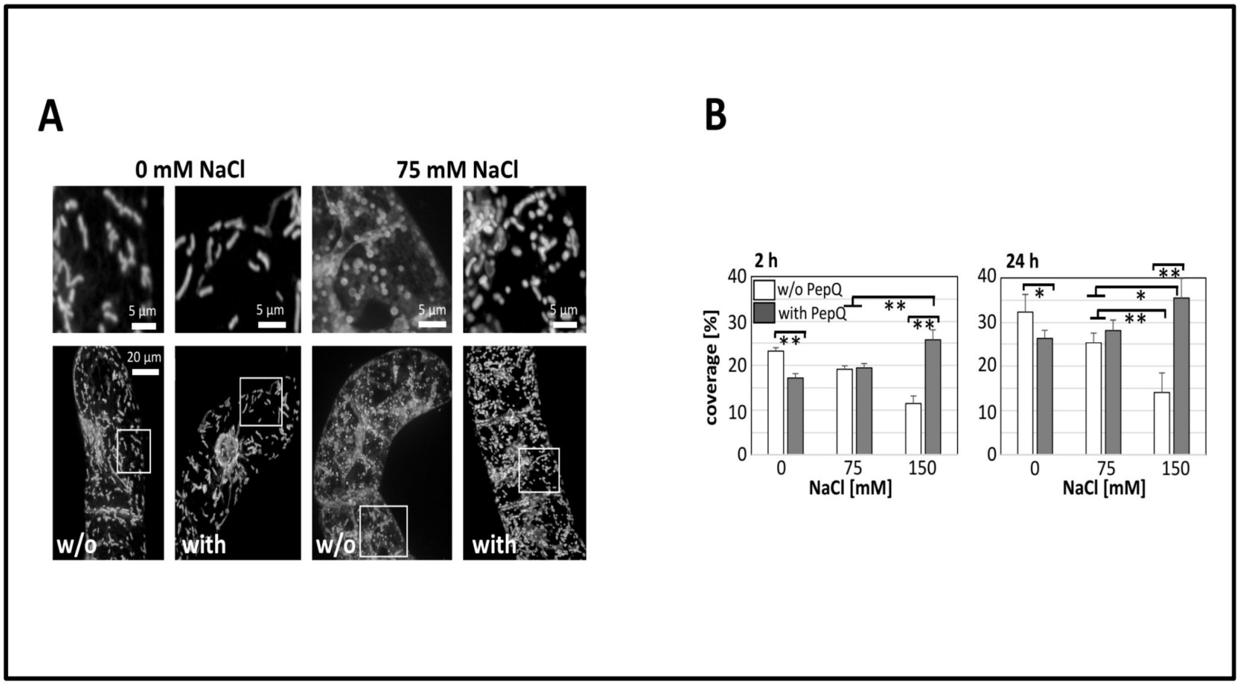


**Supplementary Figure S3**: Effect of the plant PeptoQ on mitochondrial morphology and area coverage. **A** Mitochondrial morphology in controls (0 mM NaCl) versus moderate salt stress (75 mM NaCl) in the absence (w/o), or presence (with) of plant PeptoQ (2 µM) 2 h after the onset of salt stress. The insets are given as zoom-ins in the upper row to show the differences in mitochondrial shape. **B** Mitochondrial coverage after 2 h (left), or 24 h (right) of treatment with different concentrations of NaCl. Data represent mean values and standard errors of three independent experimental series. ** indicate differences significant at $P \leq 0.01$ based on a t-test.

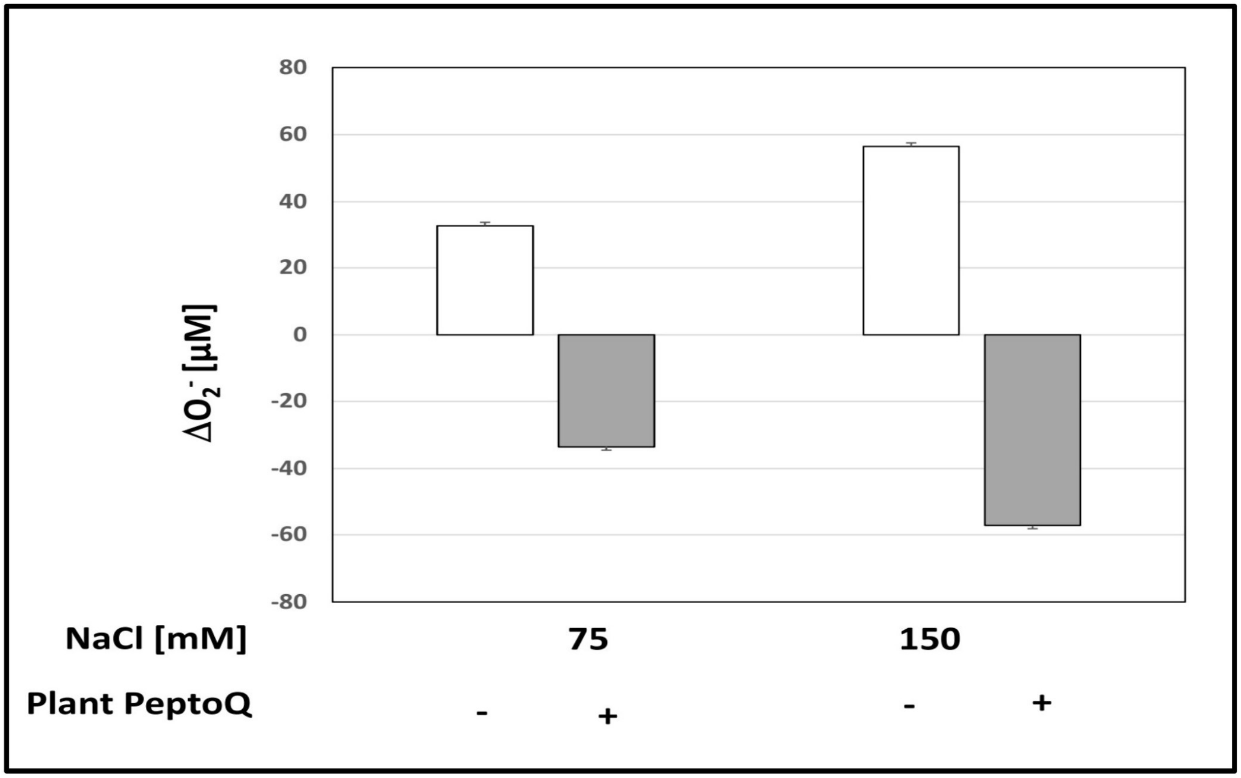


**Supplementary Figure S4:** Modulation of steady-state superoxide levels in response to salt stress tobacco BY-2 cells in absence or presence of plant PeptoQ. Plant PeptoQ was added 2 h before the onset of salinity stress. Superoxide levels were measured 4 h later. Data represent mean values and standard errors from three independent experiments.
